# Preoperative Evaluation of Soleal Vein Diameter by Ultrasound Is Beneficial for Prophylaxis of Deep Vein Thrombosis after Total Knee or Hip Arthroplasty

**DOI:** 10.1155/2018/3417648

**Published:** 2018-08-19

**Authors:** Yao Yao, Liang Qiao, Kai Song, Xingquan Xu, Dongquan Shi, Zhihong Xu, Jin Dai, Jianghui Qin, Dongyang Chen, Qing Jiang

**Affiliations:** ^1^Department of Sports Medicine and Adult Reconstructive Surgery, Drum Tower Hospital, School of Medicine, Nanjing University, Zhongshan Road 321, Nanjing, Jiangsu 210008, China; ^2^Joint Research Center for Bone and Joint Disease, Model Animal Research Center (MARC), Nanjing University, Nanjing, Jiangsu 210093, China

## Abstract

**Purpose:**

The purpose of this retrospective study was to determine the association between preoperative soleal vein (SV) diameter and deep vein thrombosis (DVT) following total joint arthroplasty (TJA).

**Methods:**

A total of 402 patients were enrolled, with 229 patients undergoing total knee arthroplasty (TKA) and 173 patients undergoing total hip arthroplasty (THA). Ultrasonography was performed 1-3 days preoperatively, on postoperative days 1, 3, and 7, or before discharge and during follow-up. The SV diameter was assessed preoperatively. Univariate analysis and multivariate logistic regression analysis were used to compare parameters between the DVT group and the non-DVT group.

**Results:**

78 patients (19.4%) were found to have DVT during hospitalization and after discharge and 14 patients (3.5%) developed symptomatic DVT. Multiple regression analysis showed that SV diameter, advanced age, and surgery type were independent predictors of postoperative DVT. In the logistic regression analysis for symptomatic DVT, SV diameter was found to be the only predictor. In the receiver operating characteristics (ROC) analysis for the value of SV diameter in diagnosing DVT, the area under curve (AUC) was 0.701 ((95% CI 0.636-0.766), P<0.001) and when the cut-off value of SV diameter was set at 0.57 cm, the sensitivity and specificity of SV diameter were 62.7% and 72.1%, respectively.

**Conclusions:**

SV diameter was an independent risk factor for total and symptomatic DVT after TJA. Preoperative ultrasound screening of the SV diameter may be beneficial for the prevention of postoperative DVT.

## 1. Introduction

Venous thromboembolism (VTE) disease is an important healthcare concern, resulting in severe life-threatening complications and healthcare resource expenditures for patients during hospitalization. Total joint arthroplasty (TJA) is a form of high-risk postoperative VTE and, without thromboprophylaxis, the rates of documented deep vein thrombosis (DVT) may range from 41 to 85% in total knee arthroplasty (TKA) and 42 to 57% in total hip arthroplasty (THA) [1]. According to the guideline, identifying patients who are at increased risk of postoperative VTE is an important step toward establishing an effective prophylactic strategy [1].

**Figure 1:**
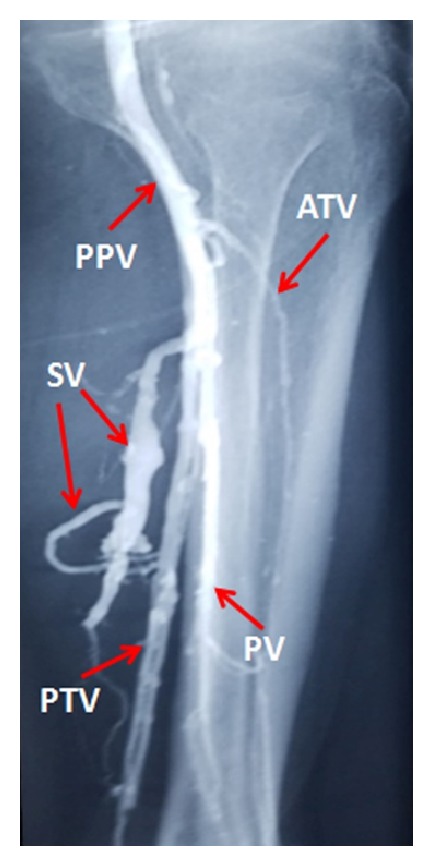
Lateral radiograph of soleal veins by venography. SV, soleal vein; PPV, popliteal vein; ATV, anterior tibial vein; PTV, posterior tibial vein; PV, peroneal vein.

**Figure 2:**
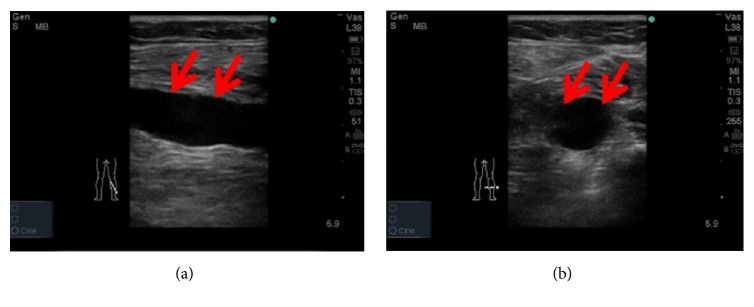
Ultrasound screening of dilated soleal vein (SV). (a) shows hypoechoic signals in the longitudinal sections of calf on B-mode screening (red arrows); (b) shows a round hypoechoic area was apparent on the transverse section (red arrows).

Soleal veins (SVs) are storage veins located inside the calf muscle that primarily drain into the posterior tibial and fibular veins [2] (Figure 1). Clinicians consider SVs to be critical in the origination of DVT and pulmonary embolism (PE) [2–6]. The high incidence of VTE in the SV may be due to its anatomical characteristics, including its high dilatation capability, which can be reliably detected by sonography [3, 5] (Figures 2(a) and 2(b)). Therefore, the preoperative evaluation of the SV may be beneficial for predicting postoperative DVT after TJA. However, to our knowledge, few studies have focused on the association between the SV diameter and postoperative DVT in patients undergoing TJA [6].

Therefore, the purpose of the present study was to test the hypothesis that the preoperative evaluation of the SV diameter by ultrasound can predict postoperative total and symptomatic DVT in TJA patients.

## 2. Material and Methods

This study was approved by the institutional ethics committee of hospital, and informed consent was obtained from all enrolled patients. Patients who received primary TKA and THA procedure were included in this study. The exclusion criteria were as follows: (1) the presence of severe liver, renal, heart, or other organs insufficiency; (2) previous VTE episode or any anticoagulant treatment [7, 8]. Finally, from March 2015 to July 2017, a total of 402 subjects who underwent primary TKA and THA at our center were enrolled. There were 173 patients undergoing THA (97 patients with osteonecrosis of the femoral head, 37 with hip osteoarthritis, 33 with acetabular dysplasia, 3 with hip rheumatoid arthritis, and 3 with ankylosing spondylitis) and 229 patients undergoing TKA (214 patients with knee osteoarthritis, 13 with knee rheumatoid arthritis, and 2 with traumatic arthritis).

The patients' medical information was recorded and evaluated before discharge, including age, gender, body mass index (BMI), history of active cancer, hypertension, diabetes mellitus, stroke, or heart disease, smoking history, surgery type (TKA/THA), postoperative pharmacological prophylaxis, operation time, transfusion status, first ambulation time (≤2d), red blood cell (RBC) count, PLT (Platelet), prothrombin time (PT), International Normalized Ratio (INR), activated partial thromboplastin time (APTT), thrombin time (TT), and levels of hemoglobin (Hb), D-Dimer, fibrinogen (Fbg), and triglyceride (TG).

## 3. Duplex Ultrasound and Postoperative Investigation

A standard ultrasound protocol was used to evaluate the bilateral lower limbs 1-3 days preoperatively by two highly skilled sonographers. Both of them have finished at least 2000 cases of ultrasound screening training for exclusion of DVT in orthopedic patients per year. A color duplex scanner (SonoSite M-Turbo) with a 5- to 10-MHz transducer was used. A compression ultrasound was assessed every 1-2 cm from the inguinal ligaments to the ankle, including the proximal (femoral and popliteal) veins to the infrapopliteal (peroneal, posterior tibial, anterior tibial, and muscular) veins. The standard diagnostic criteria for DVT were (1) an absence of compressibility of the venous segment and (2) a lack or abnormal flow on spectral analysis and color Doppler.

**Figure 3:**
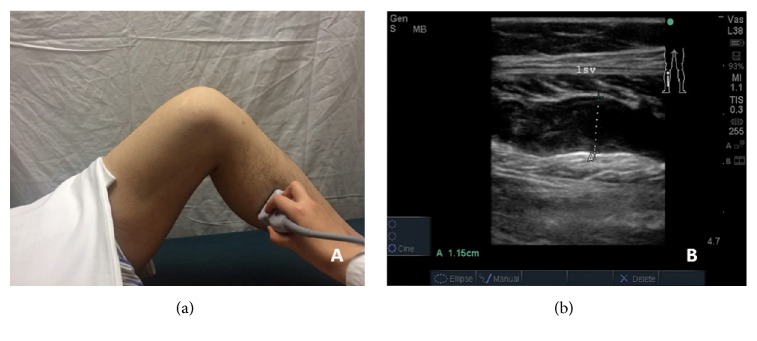
The evaluation of preoperative soleal vein (SV). (a) shows the maximum diameter (cm) of the SV was examined in a supine position with the knee flexed; (b) shows one patient has a SV diameter of 1.15 cm before TKA.

For patients without DVT, further evaluation of the SV was then conducted. The maximum diameter (cm) of the SV was examined in a supine position with the knee flexing. To avoid the misdiagnosis, routine longitude and transverse view were performed along the soleal and gastrocnemius muscle. Once the largest SV was determined, the maximal diameter was measured (Figures 3(a) and 3(b)).

Postoperative ultrasound was routinely performed on postoperative days 1, 3, and 7 (or before discharge). After discharge, patients were telephoned within three months postoperatively and were asked to return for a routine follow-up examination.

### 3.1. Surgery Information, Prophylaxis, and Management of Postoperative DVT

All surgeries were performed by 3 experienced surgeons. TKA was performed under general anesthesia with the middle surgical approach and a tourniquet. For the THA, the Hardinge approach was used for 119 patients, a modified Watson-Jones approach for 32 patients, the direct anterior approach (DAA) for 20 patients, and the SuperPath (modified microsuperior percutaneously assisted total hip) approach for 2 patients.

All patients received the recommended prophylactic anticoagulation after surgery (rivaroxaban or low molecular weight heparin), except those with a high risk of bleeding postoperatively. Rivaroxaban was taken orally at a dose of 10 mg once daily; low molecular weight heparin was injected subcutaneously at a dose of 0.4 ml per day. Thus, BMI was not taken into account for thromboprophylaxis dosage. At the same time, a compressible limb sleeve system was used after surgery for at least 8 hours per day, and a unified rehabilitation program was supervised by a physical therapist after surgery.

Once patients were diagnosed with DVT, they were given additional anticoagulants according to the site of the DVT and reassessed by duplex ultrasound during the 3 months of follow-up. Symptomatic DVT was defined as leg pain, swelling, and/or calf tenderness. If patients had clinical complaints such as chest pain, dyspnea, or oxygen desaturation, they were evaluated using spiral computed tomography pulmonary angiography to detect PE.

### 3.2. Evaluation of Risk Factors for DVT

To evaluate risk factors for total and symptomatic DVT, parameters such as age, gender, BMI, history of active cancer, hypertension, diabetes mellitus, history of stroke, smoking history, heart disease, SV diameter, surgery type, first ambulation time, postoperative pharmacological prophylaxis, operation time, transfusion, RBC, Hb, PLT, D-Dimer, PT, INR, APTT, TT, Fbg, and TG were compared.

### 3.3. Statistical Analyses

All data were analyzed using SPSS statistical software 22.0 (USA). The means were calculated to describe the continuous variables. Continuous variables (age, BMI, SV diameter, operative time, RBC, Hb, PLT, D-Dimer, PT, INR, APTT, TT, Fbg, and TG) were evaluated using the* t-*test. Categorical variables (gender, history of active cancer, hypertension, diabetes mellitus, history of stroke, smoking history, heart disease, surgery type, first ambulation time, and postoperative pharmacological prophylaxis) were compared by using the *χ*^2^ test. Binary logistic regression analysis was also used to determine the predictors for postoperative total and symptomatic DVT. Receiver operating characteristics (ROC) curve was used to determine the value of SV diameter in predicting DVT. A probability of* P<*0.05 was considered statistically significant.

## 4. Results

A total of 402 patients were enrolled in this study. There were 110 males and 292 females, with an average age of 63.55±11.25 years (range 21-85 years). The mean BMI was 25.24±3.95 kg/m^2^ (range 15.21-40.79 kg/m^2^). In total, 229 patients underwent TKA, with an average age of 67.62±8.15 years and an average BMI of 25.95±3.95 kg/m^2^. Furthermore, 173 patients underwent THA, with an average age of 58.12±12.48 years and an average BMI of 24.31±3.76 kg/m^2^. The average SV diameter was 0.56±0.24 cm (range 0.12-1.83 cm). The basic characteristics of the patients are summarized in Table 1.

### 4.1. Incidence of Postoperative DVT

**Figure 4:**
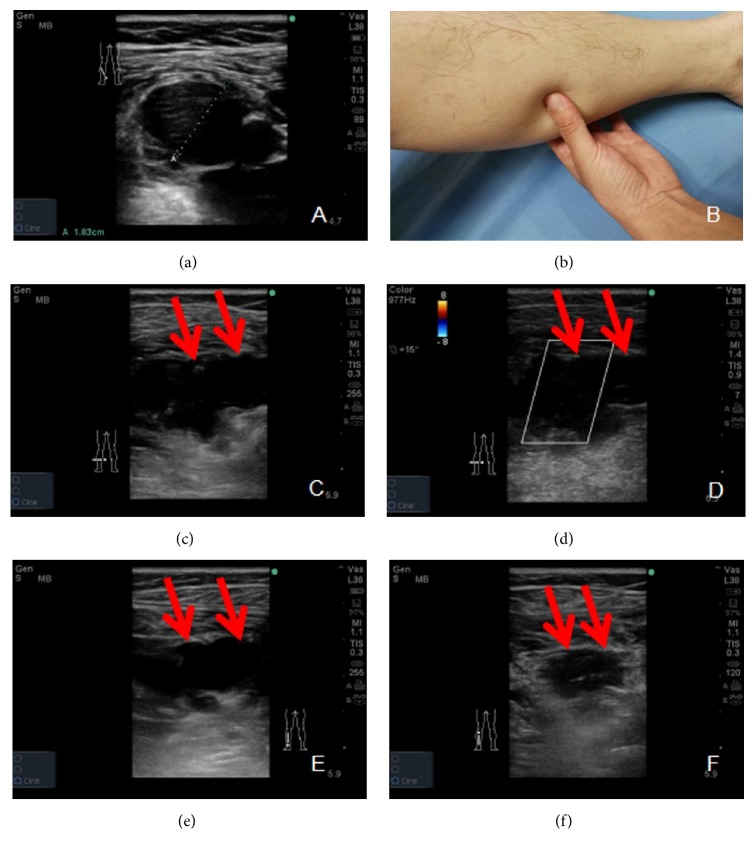
(a–f) A symptomatic deep vein thrombosis (DVT) patient with great preoperative soleal vein (SV) diameter. (a) shows that preoperative SV diameter was 1.83 cm before total hip arthroplasty; (b) shows that, 3 weeks after THA, this patient got back to hospital and presented that he could not walk normally with severe calf pain and physical examination showed homans and neuhof sign were positive; (c-d) the ultrasound screening showed a hypoechoic signals in the transverse view (c) and little blood flow signal can be visualized on color Doppler mode (d) (red arrows); (e) shows, 6 weeks after THA, B-mode shows hypoechoic areas still cannot be compressed (red arrows); (f) shows, 12 weeks after THA, the size of DVT became smaller but still existed (red arrows).

In total, 78 patients (19.4%) had postoperative DVT. Of them, 14 (3.5%) developed symptomatic DVT, and 64 (15.9%) developed asymptomatic DVT. Furthermore, 64 of the cases of DVT (27.8%) occurred after TKA, and 14 cases (8.1%) occurred after THA. In addition, 71 (91.0%) of the DVT cases occurred in the operated leg, and 7 (9.0%) cases showed bilateral DVT. All patients received a telephone interview and call back for a follow-up ultrasound screening, and 339 (84.3%) of the patients participated in the follow-up ultrasound screening (Figures 4(a)–4(f)). In addition, no cases of symptomatic PE occurred during the follow-up period. The anatomical distribution of DVT is shown in Table 2.

### 4.2. Risk Factors for Total and Symptomatic DVT after TJA

Table 3 shows the results of the univariate analysis of risk factors (general characteristics, surgical records, and laboratory tests) between the DVT group and the non-DVT group and the symptomatic DVT group and without-symptomatic DVT group. According to the univariate analysis, older age (P<0.001), larger SV diameter (P<0.001), female gender (P=0.003), a history of stroke (P=0.012), TKA surgery type (P<0.001), RBC count (P=0.001), and Hb levels (P=0.002) were significantly correlated with postoperative total DVT. Older age (P = 0.026), larger SV diameter (P=0.002), and TKA surgery (P = 0.006) were associated with symptomatic DVT.

In multiple regression analysis (Table 4), older age (OR = 1.040, P=0.024), SV diameter (OR = 10.014, P<0.001), and surgery type (OR = 0.424, P = 0.013) were found to be independent predictors of postoperative total DVT. While SV diameter (OR = 10.273, P=0.017) was found to be the only independent predictor of postoperative symptomatic DVT.

## 5. Discussion

In the present study, we determined the relationship between preoperative SV diameter and postoperative DVT after TJA. The results showed that a large SV diameter** (**OR: 10.014, 95% CI: 3.167-31.657, P<0.001**)** was significantly associated with postoperative total DVT after TJA. Moreover, the results also showed that a large SV diameter** (**OR: 10.273, 95% CI: 1.515-69.669, P=0.017**)** was significantly associated with symptomatic DVT after TJA.

To our knowledge, several studies have assessed the association between venous diameter and DVT [6, 9–12]. One study by Yamaki T et al. [11] found that a larger diameter of the gastrocnemius vein was significantly associated with postoperative DVT in TJA patients, and they concluded that a cut-off diameter value larger than 0.25 cm for the gastrocnemius vein predicted postoperative DVT. Another study from Chen et al. [9] that included 1,461 patients who underwent unilateral varicose vein surgery defined dilatation of the gastrocnemius vein as a diameter larger than 5 mm or 1.5 times the size of the normal side. The authors confirmed that the gastrocnemius vein diameter had the highest predictive power for postoperative DVT. Ogata et al. [12] measured the maximum diameter of the posterior tibial veins and peroneal veins on the paralytic side in patients with intracerebral hemorrhage and found that the maximum calf vein diameter at 2 weeks was significantly greater in patients with DVT than in patients without DVT. Unlike previous studies, we chose to measure the SV preoperatively to determine its predictive value for postoperative DVT. Our previous study [13] showed that the SV is the most common site of postoperative DVT after TJA; therefore, we speculated that a preoperative evaluation of the SV might be more valuable for predicting the formation of postoperative DVT. As expected, the results were in accordance with our prediction, and most of the cases of DVT occurred in the SVs. Our conclusion is similar to that of Abe et al. [6], who also evaluated the SV diameter in patients before major orthopedic surgery using sonography and found that a SV diameter greater than 10 mm was an independent predictor of DVT in both THA and TKA patients. We suggest that evaluating the diameter of the SV preoperatively may be beneficial for assessing the risk of postoperative DVT.

We also found that TKA has a higher association with postoperative DVT than does THA in the logistic regression analysis. Differences in risk of VTE between TKA and THA have been compared in several studies [6–8, 14, 15]. A possible explanation for the relative high incidence of DVT after TKA may be the use of a tourniquet, which may cause increased blood stasis [7]. In the study of Gionis et al., they found hypercoagulability can be documented several hours after TKA but not THA, which suggested that TKA had a stronger prothrombotic effect than THA [7]. Older age is another risk factor for postoperative DVT. The role of age in the development of DVT has not been fully elucidated in previous studies but may be attributed to immobility, increased comorbidities, decreased muscle strength of the lower extremities, endothelial dysfunction, and venous insufficiency [16].

The mechanism behind increased dilatation of the SV is unclear but may be attributed to the intrinsic nature of the SV, particularly in older patients. Some studies of the anatomy of intramuscular soleus veins have described them as having thin walls, profuse valves, and a capability for great dilatation [17, 18]. Another reason for increased dilatation is a decrease in the calf muscle. Venous reflux of the SV is highly dependent on the calf muscle pump, particularly the activation of the soleal muscle. Once the soleal muscle loses its normal function, as in the case of patients who are bedridden or immobilized, venous stasis and expansion of the SV can easily occur.

Our results showing the relationship between SV diameter and the formation of DVT might have important clinical implications for VTE prophylaxis. In the present study, chemoprophylaxis combined with mechanical prophylaxis was used during the early period. However, most DVT still occurred during the first 3 days, which is in line with previous studies [19, 20]. These data indicate that prophylactic strategies during the early period should be improved. Therefore, the evaluation of the SV diameter may be valuable as a prophylaxis for DVT and may represent a beneficial personalized preventive measure for VTE that might be recommended for specific patients. Furthermore, it is still unknown whether SV diameter is associated with DVT in other department patients. And it would be very interesting to investigate if this applies also to isolate DVT or DVT after other major operations in the future study.

The strength of this study was that we performed a routine ultrasound screening during the follow-up visit. Moreover, we enrolled a relatively large number of patients compared with previous studies. However, there are also some limitations to our study. First, we used duplex ultrasound to detect DVT in this study. Compared with contrast venography, the role of duplex ultrasound as a screening tool especially in asymptomatic patients is still controversial. However, venography is invasive, expensive, and inconvenient. As in the present study, one patient underwent ultrasound screening four times, and the routine use of venography can increase the patient's economic and physical burden. Thus, we believe that the use of ultrasound was more suitable in this study. Second, we only assessed the SV diameter in the operated leg. Whether the preoperative SV diameter is also associated with DVT in the nonoperated leg is unknown and should be evaluated in a future study. Third, this was a retrospective study and a small proportion of the patients were lost to follow-up. Thus, a large scale prospective study may make the result more convincing.

## 6. Conclusion

In conclusion, our study shows that the SV diameter, older age, and surgery type are independent predictors of postoperative DVT after TJA. Moreover, SV diameter was also associated with postoperative symptomatic DVT. Thus, a preoperative evaluation of the SV before TJA may be beneficial for prophylaxis of postoperative DVT.

## Figures and Tables

**Table 1:** Summary of patient characteristics.

Variables	No. of patients (N=402)
Age (years) (mean±SD)	63.55±11.25
Gender (female)	292 (72.6%)
BMI (kg/m^2^) (mean±SD)	25.31±4.31
Hypertension	181 (45.0%)
Diabetes mellitus	66 (16.4%)
Varicose veins	9 (2.2%)
Soleal vein diameter (cm) (mean±SD)	0.56±0.22
Malignancy	11 (3.4%)
Heart disease	46 (11.4%)
Smoking history	36 (9.0%)
Surgery type (TKA)	230 (57.2%)
Stroke	49 (12.2%)

BMI, body mass index; SD, standard deviation; TKA, total knee arthroplasty.

**Table 2:** Distribution of DVT after TJA.

Distribution	Day 1	Day 3	Day 7 or before discharge	After discharge
PV	-	-	1	-
PV+SV	2	-	-	-
PTV	1			
PTV+SV	1	-	-	-
SV	20	17	17	19
Total No. of DVT (N=78)	24	17	18	19

DVT, deep vein thrombosis; PV, peroneal vein; SV, soleal vein; PTV, posterior tibial vein.

**Table 3:** Comparison of the risk factors for TJA patients.

Variables	All patients	DVT group	Non-DVT group	P-value	Symptomatic DVT	Non- symptomatic DVT group	P-value
	N= 402	N= 78	N= 324		N=14	N=388	
Age (years)	63.55±11.25	68.50±7.97	62.36±11.6	**P<0.00**1^**∗**^	68.14±6.93	63.39±11.34	**P=0.02**6^**∗**^
Gender (female)	324 (80.6%)	67 (85.9%)	225 (77.1%)	**P=0.00**3^**∗**^	11 (78.6%)	281 (72.4%)	P=0.840
BMI (kg/m^2^)	25.32±4.31	25.99±3.85	25.15±4.40	P=0.121	25.67±4.85	25.31±4.30	P=0.758
Hypertension	181 (45.0%)	41 (52.6%)	140 (43.2%)	P=0.136	6 (42.9%)	175 (45.1%)	P=0.868
Diabetes mellitus	66 (16.4%)	10 (12.8%)	56 (17.3%)	P=0.339	1 (7.1%)	65 (16.8%)	P=0.558
Heart disease	46 (11.4%)	12 (15.4%)	34 (10.5%)	P=0.223	0	46 (11.9%)	P=0.346
Stroke	49 (12.2%)	16 (20.5%)	33 (10.2%)	**P=0.01**2^**∗**^	3 (21.4%)	46 (11.9%)	P=0.509
Malignancy	13 (3.2%)	0	13 (4.0%)	P=0.149	0	13 (3.4%)	P=1.000
Smoking history	36 (9.0%)	6 (7.7%)	30 (9.3%)	P=0.663	1 (7.1%)	35 (9.0%)	P=1.000
Varicose veins	9 (2.2%)	2 (2.6%)	7 (2.2%)	P=1.000	1 (7.1%)	8 (2.1%)	P=0.732
Soleal vein diameter (cm)	0.56±0.24	0.70±0.27	0.53±0.21	**P**<**0.00**1^**∗**^	0.76±0.36	0.56±0.23	**P=0.00**2^**∗**^
Surgery type (TKA)	230 (57.2%)	64 (82.1%)	166 (51.2%)	**P<0.00**1^**∗**^	13 (5.7%)	217 (55.9%)	**P=0.00**6^**∗**^
Postoperative pharmacological prophylaxis	391 (97.3%)	77 (98.7%)	314 (96.9%)	P=0.624	14 (100%)	377 (97.2%)	P=1.000
Operation time (min)	115±30	112.87±24.51	112.85±30.2	P=0.996	110.79±29.538	112.93±29.02	P=0.786
Transfusion	43 (10.7%)	5 (6.4 %)	38 (11.7%)	P=0.172	2 (14.3%)	41 (10.6%)	P=0.998
First ambulation time (≤2d)	232 (57.7%)	45 (57.7%)	187 (57.7%)	P=0.997	3 (21.4%)	167 (43.0%)	P=0.108
RBCs	4.3±0.4	4.17±0.36	4.35±0.44	**P<0.00**1^**∗**^	4.10±0.26	4.33±0.44	P=0.058
Hb (g/dL)	129±15	124.56±12.24	130.30±14.93	**P=0.00**2^**∗**^	122.21±13.66	129.44±14.60	P=0.069
PLT (10^3^/mL)	206±61	211.60±59.72	206.78±59.82	P=0.523	190.21±40.0	208.35±60.14	P=0.265
D-Dimer (mg/mL)	0.92±1.44	1.30±2.16	0.82±1.19	P=0.062	0.84±0.85	0.91±1.46	P=0.846
PT (S)	11.99±0.98	11.77±0.81	11.93±0.98	P=0.180	27.25±4.80	28.07±5.77	P=0.702
INR	1.04±0.09	1.02±0.07	1.04±0.10	P=0.162	1.04±0.08	1.03±0.09	P=0.748
APTT (S)	28.16±6.06	27.11±3.54	28.27±6.13	P=0.111	27.25±4.80	28.07±5.77	P=0.599
TT (S)	19.56±14.90	20.54±21.29	19.25±11.16	P=0.447	18.49±1.28	19.54±13.64	P=0.774
Fbg (g/L)	2.94±0.80	2.87±0.76	2.99±0.83	P=0.227	2.58±0.50	2.98±0.82	P=0.068
TG (mmol/L)	1.51±0.68	1.52±0.66	1.48±0.73	P=0.649	1.40±1.02	1.49±0.70	P=0.638

DVT, deep vein thrombosis; BMI, body mass index; TKA, total knee arthroplasty; RBCs, red blood cells; Hb, hemoglobin; PT, prothrombin time; INR, International Normalized Ratio; APTT, activated partial thromboplastin time; TT, thrombin time; Fbg, fibrinogen; TG, triglyceride.

^**∗**^P<0.05 was considered statistically significant.

**Table 4:** Logistic regression analysis to identify the independent risk factors for total and symptomatic DVT.

Variables	Risk factors for total DVT			Risk factors for symptomatic DVT		
	*P*-value	OR	95% CI	*P*-value	OR	95% CI
Age	0.024^*∗*^	1.040	1.005-1.076	0.802	1.009	0.941-1.081
Gender	0.233	0.622	0.285-1.358	-	-	-
Stroke	0.238	1.536	0.753-3.132	-	-	-
SV diameter	0.000^*∗*^	10.014	3.167-31.657	0.017^*^	10.273	1.515-69.669
Surgery type (TKA)	0.013^*∗*^	0.424	0.215-0.837	0.064	0.133	0.016-1.121
Red blood cells	0.15	0.433	0.138-1.354	-	-	-
Hemoglobin	0.819	0.996	0.961-1.032	-	-	

OR, odds ratio; CI, confidence interval; SV, soleal vein; TKA, total knee arthroplasty.

^*∗*^P<0.05 was considered statistically significant.

## Data Availability

The data used to support the findings of this study are available from the corresponding author upon request.

## Abbreviations

SV: Soleal vein
TJA: Total joint arthroplasty
THA: Total hip arthroplasty
TKA: Total knee arthroplasty
THA: Total hip arthroplasty
DVT: Deep vein thrombosis
ROC: Receiver operating characteristics
AUC: Area under curve
CI: Confidence interval
OR: Odds ratio
VTE: Venous thromboembolism
PE: Pulmonary embolism
BMI: Body mass index
RBC: Red blood cell
PLT: Platelet
PT: Prothrombin time
INR: International Normalized Ratio
APTT: Activated partial thromboplastin time
TT: Thrombin time
Hb: Hemoglobin
Fbg: Fibrinogen
TG: Triglyceride
DAA: Direct Anterior Approach
Superpath: Supercapsular Percutaneously Assisted Total hip
SD: Standard deviation
PPV: Popliteal vein
PV: Peroneal vein
ATV: Anterior tibial vein
PTV: Posterior tibial vein.

## References

[1] Geerts W. H., Pineo G. F., Heit J. A. (2004). Prevention of venous thromboembolism: the seventh ACCP conference on antithrombotic and thrombolytic therapy. *CHEST*.

[2] Kageyama N., Ro A., Tanifuji T. (2008). Significance of the soleal vein and its drainage veins in cases of massive pulmonary thromboembolism. *Annals of Vascular Diseases*.

[3] Ohgi S., Tachibana M., Ikebuchi M., Kanaoka Y., Maeda T., Mori T. (1998). Pulmonary embolism in patients with isolated soleal vein thrombosis. *Angiology*.

[4] Ro A., Kageyama N. (2016). Clinical significance of the soleal vein and related drainage veins, in calf vein thrombosis in autopsy cases with massive pulmonary thromboembolism. *Annals of Vascular Diseases*.

[5] Ohgi S., Ohgi N. (2014). Relationship between specific distributions of isolated soleal vein thrombosis and risk factors. *Annals of Vascular Diseases*.

[6] Abe K., Yuda S., Yasui K. (2017). Soleal vein dilatation assessed by ultrasonography is an independent predictor for deep vein thrombosis after major orthopedic surgery. *Journal of Cardiology*.

[7] Gionis M. N., Ioannou C. V., Katsamouris A. N. (2013). The study of the thrombin generation mechanism and the effect of low molecular weight heparin as thromboprophylaxis in patients undergoing total knee and hip replacement. *Thrombosis Research*.

[8] Gionis M. N., Ioannou C. V., Kontopodis N., Balalis K., Elalamy I., Gerotziafas G. T. (2016). Heparin resistance and coagulation activation rebound effect after anticoagulant withdrawal: Beneficiary effect of adjuvant antiplatelet therapy. *International Angiology*.

[9] Chen K., Yu G.-F., Huang J.-Y. (2015). Incidence and risk factors of early deep venous thrombosis after varicose vein surgery with routine use of a tourniquet. *Thrombosis Research*.

[10] Na M., Jin G. F., Bin W. H. (2012). Value of color doppler ultrasonography in early diagnosis of lower limb deep vein thrombosis after surgery. *Chinese Journal of Ultrasound in Medicine*.

[11] Yamaki T., Hamahata A., Fujisawa D. (2011). Deep vein thrombosis after total knee or hip arthroplasty is associated with increased preoperative calf muscle deoxygenation as measured by near-infrared spectroscopy. *Journal of Vascular Surgery*.

[12] Ogata T., Yasaka M., Wakugawa Y., Kitazono T., Okada Y. (2013). Association of deep venous thrombosis with calf vein diameter in acute hemorrhagic stroke. *Journal of Stroke and Cerebrovascular Diseases*.

[13] Yao Y., Zhang C. J., Dai X. Y. (2013). The anatomical distribution of lower extremity deep vein thrombosis after total knee and hip arthroplasty. *Chinese Journal of Orthopaedics*.

[14] White R. H., Romano P. S., Zhou H., Rodrigo J., Bargar W. (1998). Incidence and time course of thromboembolic outcomes following total hip or knee arthroplasty. *JAMA Internal Medicine*.

[15] Bjornara B. T., Gudmundsen T. E., Dahl O. E. (2006). Frequency and timing of clinical venous thromboembolism after major joint surgery. *The Journal of Bone & Joint Surgery*.

[16] Engbers M. J., van Hylckama Vlieg A., Rosendaal F. R. (2010). Venous thrombosis in the elderly: Incidence, risk factors and risk groups. *Journal of Thrombosis and Haemostasis*.

[17] Reis F. P., Aragão J. A., de Figueiredo L. F. P., Miranda F., Nunes M. A. P., Feitosa V. L. C. (2008). Venous drainage of the soleus muscle. *Surgical and Radiologic Anatomy*.

[18] Cockett F. B. (1955). The pathology and treatment of venous ulcers of the leg. *British Journal of Surgery*.

[19] Yamaguchi T., Hasegawa M., Niimi R., Sudo A. (2010). Incidence and time course of asymptomatic deep vein thrombosis with fondaparinux in patients undergoing total joint arthroplasty. *Thrombosis Research*.

[20] Maynard M. J., Sculco T. P., Ghelman B. (1991). Progression and regression of deep vein thrombosis after total knee arthroplasty. *Clinical Orthopaedics and Related Research*.

